# Genetic diversity, population structure and relationships in indigenous cattle populations of Ethiopia and Korean Hanwoo breeds using SNP markers

**DOI:** 10.3389/fgene.2013.00035

**Published:** 2013-03-21

**Authors:** Zewdu Edea, Hailu Dadi, Sang-Wook Kim, Tadelle Dessie, Taeheon Lee, Heebal Kim, Jong-Joo Kim, Kwan-Suk Kim

**Affiliations:** ^1^International Livestock Research InstituteAddis Ababa, Ethiopia; ^2^Department of Animal Science, Chungbuk National UniversityCheongju, Korea; ^3^Department of Animal Biotechnology, College of Animal Bioscience and Technology, Konkuk UniversitySeoul, Korea; ^4^Department of Agricultural Biotechnology, Research Institute for Agriculture and Life Sciences, Seoul National UniversitySeoul, Korea; ^5^School of Biotechnology, Yeungnam UniversityGyeongsan, Korea

**Keywords:** Ethiopia, genetic diversity, population structure, SNP, Hanwoo

## Abstract

In total, 166 individuals from five indigenous Ethiopian cattle populations – Ambo (*n* = 27), Borana (*n* = 35), Arsi (*n* = 30), Horro (*n* = 36), and Danakil (*n* = 38) – were genotyped for 8773 single nucleotide polymorphism (SNP) markers to assess genetic diversity, population structure, and relationships. As a representative of taurine breeds, Hanwoo cattle (*n* = 40) were also included in the study for reference. Among Ethiopian cattle populations, the proportion of SNPs with minor allele frequencies (MAFs) ≥0.05 ranged from 81.63% in Borana to 85.30% in Ambo, with a mean of 83.96% across all populations. The Hanwoo breed showed the highest proportion of polymorphism, with MAFs ≥0.05, accounting for 95.21% of total SNPs. The mean expected heterozygosity varied from 0.370 in Danakil to 0.410 in Hanwoo. The mean genetic differentiation (*F*_ST_; 1%) in Ethiopian cattle revealed that within individual variation accounted for approximately 99% of the total genetic variation. As expected, *F*_ST_ and Reynold genetic distance were greatest between Hanwoo and Ethiopian cattle populations, with average values of 17.62 and 18.50, respectively. The first and second principal components explained approximately 78.33% of the total variation and supported the clustering of the populations according to their historical origins. At *K* = 2 and 3, a considerable source of variation among cattle is the clustering of the populations into Hanwoo (taurine) and Ethiopian cattle populations. The low estimate of genetic differentiation (*F*_ST_) among Ethiopian cattle populations indicated that differentiation among these populations is low, possibly owing to a common historical origin and high gene flow. Genetic distance, phylogenic tree, principal component analysis, and population structure analyses clearly differentiated the cattle population according to their historical origins, and confirmed that Ethiopian cattle populations are genetically distinct from the Hanwoo breed.

## INTRODUCTION

Ethiopia, with its 49.33 million heads of cattle, has the largest cattle population in Africa (Central Statistical Authority (CSA), 2008). The biological diversity of indigenous cattle populations/breeds is a key to sustaining the wellbeing of millions of farming and pastoral communities, predominantly inhabiting low-input production systems. Geographical proximity to the entry points of Indian and Arabian zebu (*Bos indicus*) and the Near Eastern *B. taurus* (Rege, 1999; Hanotte et al., 2002) offered an opportunity for multiple livestock introduction into East Africa. Ethiopia is home to over 24 cattle breeds or populations, which can be grouped into four categories: zebu (*B. indicus*), sanga (zebu × *B. taurus*), zenga (sanga × zebu), and the humpless *B. taurus* (Rege, 1999). Many of them are named after the community, which keeps the population, or according to geographical localities they inhabit, and the true genetic relationship between the major populations is not yet well known or documented.

There has been a rapid decline in population and identity of most indigenous cattle populations of East Africa through breed substitution, indiscriminate crossbreeding, the absence of breed development programs, and environmental changes (Rege and Gibson, 2003; Hanotte et al., 2010). For instance, of the 145 breeds identified in sub-Saharan Africa, 47 (approximately 32%) were considered to be at a risk of extinction, and in total, 22 breeds (approximately 13%) previously recognized in the continent have become extinct in the last century (Rege, 1999). The erosion of locally adapted genetic resources will significantly limit the option and capacity to cope with changes to production environments and breeding goals. Understanding of farm animal genetic diversity is therefore required to contribute to meeting current production needs in various environments, to allow sustained genetic improvement, and to facilitate rapid adaptation to changing environments and breeding objectives (Notter, 1999; Köhler-Rollefson et al., 2009; Hanotte et al., 2010).

Previous studies focused on genetic diversity and structures of Ethiopian cattle populations have used low-density microsatellite, mitochondrial, or Y-chromosome markers (Li et al., 2007; Dadi et al., 2008, 2009; Zerabruk et al., 2011). However, in recent years, analyses of single nucleotide polymorphism (SNP) markers are becoming the standard approach for diversity analysis and genome-wide studies. They represent one of the more interesting approaches for genotypization because they are abundant in the genome, genetically stable, and amenable to high-throughput automated analysis (Vignal et al., 2002). The usefulness of SNPs in analyses of population diversity and structure has been demonstrated in several studies (McKay et al., 2008; Lin et al., 2010). The identification of genomic SNPs will provide an opportunity to apply genome-based association studies in the future. Despite a large number of SNPs identified in the bovine genome-sequencing project, few have been validated in Ethiopian cattle populations. Breed characterization requires basic knowledge of genetic variations that can be effectively measured within and between populations. The present study was thus undertaken to analyze the level of genetic diversity, population structure, and relationships between five indigenous Ethiopian cattle populations, and the Hanwoo breed, using 4235 autosomal genome-wide SNPs.

## MATERIALS AND METHODS

### BREEDS AND DNA SAMPLE COLLECTION

**FIGURE 1:**
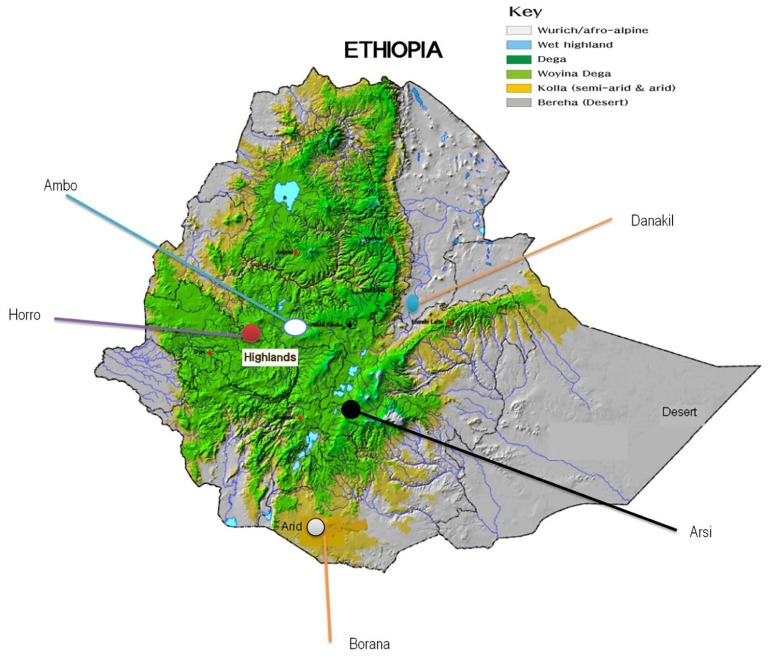
**Geographical locations of the five Ethiopian cattle populations sampled (Edea et al., 2012)**.

Nasal samples were collected from a total of 166 randomly selected animals representing five indigenous Ethiopian cattle populations: Ambo (*n* = 27), Borana (*n* = 35), Arsi (*n* = 30), Horro (*n* = 36), and Danakil (*n* = 38). As a representative of taurine breed, Hanwoo cattle (*n* = 40) were also included in the study for reference. The target populations/breeds represent the four main groups of cattle: zebu (Borana, Ambo, and Arsi), sanga (Danakil), zenga (Horro; Rege, 1999), and taurine (Hanwoo). During sampling, potential geo-environmental gradients (highlands and lowlands), production systems (mixed crop-livestock and pastoral/agro-pastoral), and ethnic groups predominantly raising the populations were considered (**Table 1**; **Figure 1**). To minimize the sampling of related animals, herdsmen and owners of the animals were contacted. Nasal samples were collected using Performagene LIVESTOCK’s nasal swab DNA collection kit and DNA was extracted from nasal samples according to the manufacturer’s recommendations (DNA Genotek Inc., 2012).

**Table 1:** Summary of sampled populations and their characteristics with respect to breed group, distribution over ecological zones and affinity to ethnic communities (Edea et al., 2012).

Breed/population	Breed group	Agro-ecology	Community	Production system
Ambo	Small East African Zebu	III	Oromo	Mixed crop-livestock
Arsi	Large East African Zebu	III	Oromo	Mixed crop-livestock
Borana	Large East African Zebu	IV	Oromo	Pastoral
Danakil	Sanga	IV	Afar	Pastoral
Horro	Zenga	III	Oromo	Mixed crop-livestock

*Agro-ecologies: III Wet highland: tepid to cool wet highlands, very high vegetation, 2,091 masl, 1437 mm rain, maximum 24.8°C, minimum 10.1°C. IV. Arid lowland: hot arid lowland plain, very low vegetation, 894 masl, 404.5 mm rain, maximum 33.2°C, minimum 17.4°C(Ministry of Agriculture (MoA), 1998).*

### GENOTYPING, QUALITY CONTROL, AND MARKERS SELECTION

In total, 166 randomly sampled animals representing five indigenous cattle populations of Ethiopia were genotyped for 8,773 SNPs using the Illumina Bovine 8K SNP BeadChip (Boichard et al., 2012), which was commercially available at the GeneSeek (GeneSeek, Lincoln, NE). Over all loci, the average GenTrain score, 10% GenCall (10% GC) score, and 50% GenCall (50%GC) score were 0.86, 0.87, and 0.83, respectively. In this experiment, 99.91% of the markers identified had GenTrain scores greater than the minimum acceptable value (0.25; http://www.illumina.com). Autosomal markers (6987) were used for SNP polymorphism distribution analysis. Markers selected for diversity analysis were required to be located on autosomal chromosomes, have call rates of ≥90%, and have minor allele frequency (MAF) ≥0.05 in all populations included in the study. After application of these selection criteria, 4235 SNPs remained and were included in the analysis.

### STATISTICAL ANALYSES

#### Analyses of molecular variance and within-breed genetic diversity determination

Analyses of molecular variance (AMOVA) analyses were carried out on three datasets by using the program Arlequin (Excoffier et al., 2005). The first analysis included data for all six populations (i.e., Hanwoo and the five Ethiopian cattle populations); the second dataset consisted of the Ethiopian cattle grouped by the three breed types, zebu (Borana, Arsi, and Ambo), sanga (Danakil), and zebu × sanga (Horro); and a third analysis was performed by grouping Ethiopian cattle according to their ecological distribution (highland and lowland populations). Observed and expected heterozygosity (Nei, 1987) was estimated using the same software. Deviation from Hardy–Weinberg equilibrium (HWE; heterozygote deficiency) was assessed by performing a chi-square test with the PowerMarker program (Liu and Muse, 2005) for each marker and population.

#### Genetic differentiation and relationships among the populations

Fixation indices were estimated according to Weir and Cockerham (1984) by using the program Arlequin (Excoffier et al., 2005). The significance of fixation indices were determined using permutation tests (1000 permutations). The significance of pair-wise population differentiation values was similarly determined by permutation testing (1000 permutations). Reynolds’ genetic distance (Reynolds et al., 1983), recommended for use with populations with short divergence times, between different pairs of cattle populations was calculated using PowerMarker (Liu and Muse, 2005). Unweighted pair-group method with arithmetic mean (UPGMA; Sneath and Sokal, 1973) algorithms was used to construct the dendrogram from Reynolds’ matrices using PowerMarker (Liu and Muse, 2005). The generated tree was visualized in Mega tree explorer (Tamura et al., 2011). The effective migration rate (*N*_m_), an indirect estimate of gene flow, was calculated using the program GenAlEx 6.41 (Peakall and Smouse, 2006).

#### Principal component analysis

A principal component analysis (PCA) was carried out to illustrate the relationship among the populations by using Golden Helix SNP Variation Suite version 7 (Golden Helix, Inc., 2012). PCA was carried out to determine breed relationships based directly on allele frequencies by using a multivariate method, which condenses the information from a large number of alleles and loci in to a few synthetic variables (PCs).

#### Structure analysis

For the analysis of population structure, a Bayesian model-based analysis was performed with the most commonly used software STRUCTURE 2.3.4 (Pritchard et al., 2000). This software assumes a model in which there are *K* populations (clusters), which contribute to the genotype of each individual and each is characterized by a set of allele frequencies at each marker locus. The method attempts to assign individuals to populations on the basis of their genotypes, while simultaneously estimating progenitor population allele frequencies. A Monte Carlo Markov chain method was used to estimate allele frequencies in each of the *K* populations and the degree of admixture for each individual animal. The number of clusters was inferred using five independent runs with 100,000 iterations and a burn-in period of 20,000 following the admixture ancestry model and correlated allele frequencies with *K* values ranging from two to six. We performed four independent runs for each predefined number of populations (*K* = 2–6).

## RESULTS

### SNP POLYMORPHISM AND WITHIN POPULATION GENETIC DIVERSITY

Level of polymorphism and genetic variability within the different cattle populations and departures from the HWE are shown in **Table 2.** Among Ethiopian cattle populations, the proportion of SNPs with a MAF ≥0.05 ranged from 81.63% in Borana to 85.30% in Ambo, with a mean of 83.96% across all populations. The Hanwoo breed, where MAFs ≥ 0.05 accounted for 95.21% of all SNPs, showed the highest proportion of polymorphism. The mean expected heterozygosity varied from 0.363 (0.130) in Danakil to 0.415 (0.123) in Hanwoo. Similarly, the highest and lowest observed heterozygosity was found in the same 2 breeds (0.363 and 0.410 in Danakil and Hanwoo, respectively). The mean values of observed and expected heterozygosities in Hanwoo were generally higher than those in Ethiopian cattle populations. The majority of polymorphic SNPs were found to be in HWE, both across the whole sample and in each separate population. For Ethiopian cattle populations, only 293 (6.92%) of 4235 SNPs markers were calculated to significantly deviate from HWE (*p* ≤ 0.05), whereas in Hanwoo, 240 (5.67%) of the markers appeared to be in disequilibrium.

**Table 2:** Genetic variability within cattle populations.

Population	*N*	*H*_ob_ (SD)	*H*_ex_(SD)	Inbreeding(f)	% Markers with MAF ≥ 0.05	% SNPS not in HWE (*P* ≤ 0.05)
Horro	36	0.387 (0.115)	0.388 (0.105)	-0.012	84.84	3.02
Danakil	38	0.363 (0.130)	0.370 (0.116)	0.012	82.81	3.09
Borana	35	0.374 (0.132)	0.372 (0.114)	-0.017	81.63	2.34
Arsi	30	0.376 (0.130)	0.382 (0.109)	0.004	85.26	2.98
Ambo	27	0.386 (0.131)	0.389 (0.106)	-0.004	85.30	2.65
Hanwoo	40	0.415 (0.123)	0.410 (0.104)	-0.014	95.21	5.67

### POPULATION STRUCTURE

#### AMOVA and genetic differentiation

Analyses of molecular variance were performed to examine the partitioning of genetic variation. Analyses revealed that the five Ethiopian cattle populations had overall fixation indices of 1% (*F*_ST_), 0.6% (*F*_IT_), and -0.3% (*F*_IS_; **Table 3**). *F*_ST_ value for Ethiopian cattle population was generally very low; however, significant (*p* < 0.001), while most of the population variance could be explained by within individual variability. Within-populations inbreeding (*F*_IS_) and total inbreeding (*F*_IT_) were not found to be significant (*p* > 0.05). Further analysis of the five Ethiopian cattle populations grouped by breed types showed that within-individual genetic differences accounted for 99.44% of variation, with variation among groups being insignificant, at -0.04% (*F*_CT_; *p* > 0.05). When Hanwoo cattle were included in the analysis, the mean variance among groups was significant at 17.40% (*F*_CT_; *p* < 0.0001), while 81.51% of the total genetic variation was within populations. Grouping of Ethiopian cattle populations based on their ecological distributions (highland and lowland) revealed an estimated among-group variation of 0.42% (F_CT_).

**Table 3:** AMOVA analysis results using different data sets.

Data set	Variance component (%)			Fixation indices			
	Among groups	Among populations within groups	Within individuals	*F*_CT_	*P*value	*F*_SC_	*P*value
All six populations	17.46	0.710	81.51	0.174	0.000 ± 0.000	0.009	0.000 ± 0.000
Ethiopia cattle grouped based on breed group	-0.04	0.93	99.44	-0.0004	0.512 ± 0.014	0.009	0.000 ± 0.000
Ethiopia cattle grouped based on ecological distribution	0.407	0.64	98.16	0.004	0.000 ± 0.000	0.006	0.000 ± 0.000

**FIGURE 2:**
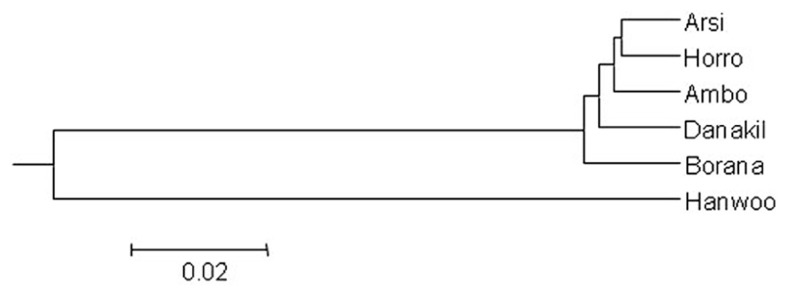
**Phylogenic tree showing the genetic relationships among the six cattle populations using Reynolds et al. (1983) genetic distance**.

For each pair of cattle populations, pair-wise population *F*_ST_ and Reynolds’s genetic distance are shown in **Table 4**. Within Ethiopian cattle populations, the lowest *F*_ST_ (0.002) and Reynold genetic distance (0.019) were both found between Horro and Ambo populations. As expected, *F*_ST_ and Reynold genetic distance were the largest between Hanwoo and Ethiopian cattle populations with average values of 17.62 and 18.50, respectively. For all pairs of populations, the *F*_ST_ statistic indicated statistically significant differences (*p* < 0.01), revealing significantly differentiated populations. The mean number of migrants per generation (*N*_m_) across all Ethiopian cattle populations was estimated at 15. The phylogenetic relationship among the six cattle populations, based on Reynolds’ distance (Reynolds et al., 1983), is depicted in **Figure 2.** As expected, the populations are clearly separated into Hanwoo and Ethiopian cattle groups. Within Ethiopian cattle populations, Arsi and Horro formed a closely related sub-cluster, whereas Borana were placed in a relatively separate group. Danakil and Ambo were intermediately positioned between the Borana and the Arsi and Horro sub-cluster.

**Table 4:** Pair-wise genetic differentiation (*F*_ST_) values between the six cattle populations (below diagonal) and Reynolds’ genetic distance (above diagonal).

Population	Ambo (AMB)	Arsi (ARS)	Borana (BOR)	Danakil (DAN)	Horro (HOR)	Hanwoo (HAN)
AMB		0.020	0.030	0.026	0.019	0.177
ARS	0.002**		0.027	0.022	0.017	0.183
BOR	0.013**	0.011**		0.028	0.028	0.193
DAN	0.010**	0.007**	0.014**		0.024	0.196
HOR	0.002**	0.002**	0.014**	0.010**		0.176
HAN	0.167**	0.173**	0.184**	0.189**	0.168**	

**P < 0.01. Significance levels were obtained after 1,000 permutations.

#### Principal component and structure analysis

**FIGURE 3:**
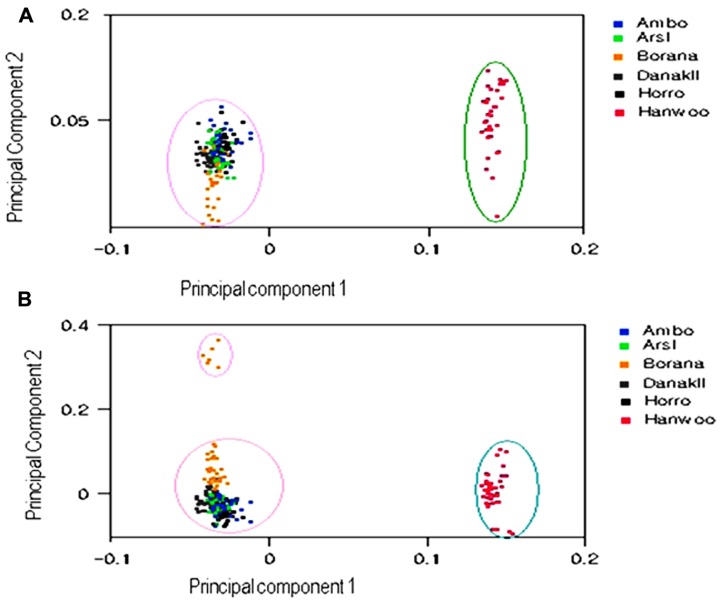
**First and second principal component (A) and first and third principal component (B) analysis results from 4235 SNPs in six cattle populations**.

**Figure 3** depicts analyses of three principal components for 4235 markers in the six cattle populations. PCA evidently distinguished Ethiopian cattle populations from Hanwoo, with the first and second principal components (PC1 and PC2) explaining 71.75% and 6.58%, respectively, of the total variation. On clustering of the populations according to their origin, PC1 and PC2 explained approximately 78.33% of the total variation, whereas the third principal component (PC3) accounted for approximately 6.21% of the variation and somewhat isolated the Borana cattle population from the other Ethiopian cattle. Further, PCA analysis validated the results of the population structure and dendrogram analysis.

**FIGURE 4:**
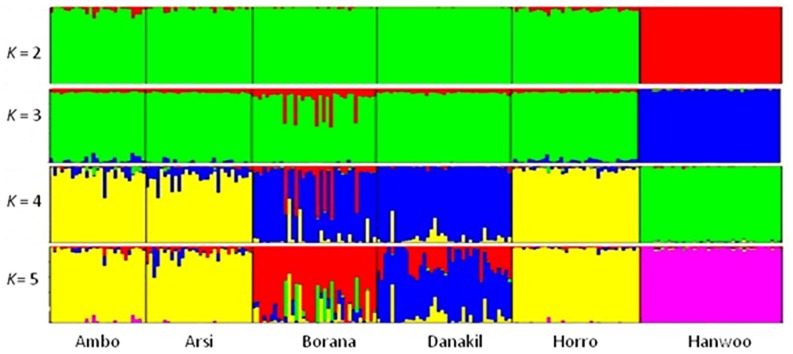
**Summary of plots of estimate of Q for *K * = 5 in six cattle populations.** Each individual is represented by a single vertical divided in to *K* colored segments, where *K* is the number of cluster assumed with the length proportional to each of the *K* inferred cluster. Black color separated the populations indicated below the figure.

A graphic representation of cluster structure analysis is depicted in **Figure 4**. At *K* = 2 and 3, a considerable source of variation among cattle is the clustering of the cattle into Hanwoo (taurine) and Ethiopian cattle populations. At *K* = 2 and 3, with the exception of approximately 12% of the individual animals from the Borana population, which clustered in separate group, there was no clear differentiation among indigenous Ethiopian cattle populations and they did not cluster according to their traditional classifications or geographical distribution. Differentiation within Ethiopian populations was first observed at *K* = 4 breeds, with over 88% of Ambo, Arsi, and Horro (highland populations) assigned to the same cluster and 77% of the Borana and 92% of Danakil (lowland breeds) sharing the same cluster, which corresponded to the ecological distribution of these populations. **Table 5** presents the proportion of the six populations belonging to each of the five clusters. At least 88% of Ambo, Arsi, and Horro cattle were assigned to cluster 4, while 71% of Danakil and 75% of Borana were in clusters 3 and 1, respectively. The results also indicate that 97% Hanwoo fall within cluster 5.

**Table 5:** Proportion of analyzed cattle breeds/populations in each of the six clusters (*K* = 5).

Population	Inferred cluster				
	1	2	3	4	5
Ambo	0.031	0.002	0.024	0.926	0.016
Arsi	0.049	0.003	0.058	0.886	0.004
Borana	0.746	0.087	0.065	0.10	0.002
Danakil	0.203	0.005	0.711	0.079	0.001
Horro	0.013	0.002	0.016	0.963	0.006
Hanwoo	0.009	0.003	0.006	0.008	0.974

## DISCUSSION

### GENETIC VARIABILITY WITHIN POPULATIONS

Most SNPs identified in the Ethiopian and Hanwoo cattle populations exhibited a high degree of polymorphism. Hanwoo cattle displayed the highest, and Borana the lowest, levels of polymorphism. The level of polymorphic SNPs in this study was higher than has previously been reported for taurine breeds (Dadi et al., 2012). Similarly, Gautier et al. (2007) reported levels of polymorphism of 93.5% in European cattle breeds and the same authors found a lower degree of SNP polymorphism in African cattle breeds (47. 4% in Lagune and 71.0% in Borgou) based on analysis of 696 SNPs. The observation of greater polymorphism in Hanwoo cattle in this study could be a reflection of the fact that most publically available bovine sequence data are from *B. taurus* breeds. *B. taurus* populations have previously been reported to have higher MAFs than *B. indicus* breeds (Lin et al., 2010). Genetic variability was highest in Hanwoo cattle (0.410), whereas among the Ethiopian cattle populations, Danakil demonstrated the lowest genetic variability (0.370). The relatively lower genetic diversity observed in the Danakil population could be due to inbreeding (*F*_IS_**= 0.012) and uncontrolled mating practices that are common among the pastoral herds. In the pastoral communal system, animals have more chance to mix both while grazing and at watering points.

Among Ethiopian cattle populations, observed and expected heterozygosity values were lower in this SNP-based study than those estimated using microsatellite markers (Dadi et al., 2008) for 10 Ethiopian cattle populations. Similarly, Carruthers et al. (2011) reported higher values based on microsatellites marker studies, than those obtained using SNP analysis, in Angus and Brazilian cattle breeds. The difference in the results obtained using these two techniques could be a reflection of the multi-allelic nature of microsatellite markers. Moreover, the results presented here are very similar to the 0.386 expected heterozygosity observed in Podolic cattle breeds by using SNPs (Pariset et al., 2010). Lower values of heterozygosity have been described for the Angus breed (0.332; Carruthers et al., 2011), and heterozygosity values (H_e_) of 0.25 for African cattle and 0.30 for European originated breeds, respectively, based on SNP analysis were reported (Gautier et al., 2007). The higher genetic variability noted in Hanwoo cattle is in harmony with the results of Lin et al. (2010), who observed lower genetic diversity in *B. indicus*, compared to *B. taurus*, breeds. By contrast, a high level of genetic diversity was observed in Eastern Africa than in Western Africa and Europe (Hanotte et al., 2002) microsatellite analysis. The differences in reported results may be explained by the application of different molecular markers.

Genetic diversity is in fact important to allow genetic improvement and facilitate rapid adaptation to changing environments and breeding objectives (Notter, 1999). The higher variability observed within Ethiopian cattle populations can potentially be attributed to the absence of strong artificial selection pressures, a high levels of admixture in these populations (Rege, 1999; Dadi et al., 2008) causing increased heterozygosity, which is a distinctive trait of large traditional populations. The introduced zebu cattle intermingled and crossbred with the original African long horn taurine population to produce the various types of cattle found in East Africa today, as has been well documented (Payne and Wilson, 1999; Hanotte et al., 2002).

### POPULATION STRUCTURE

#### AMOVA and genetic differentiation

Pair-wise population differentiation (*F*_ST_) and Reynolds’ genetic distance estimates revealed close relationships among Ethiopian cattle populations. The low level of differentiation between the Ethiopian cattle population (*F*_ST_**= 1%) could be attributed to common ancestry, short domestication history, admixture of the population, and lack of selection pressure. The value observed in this study is in good agreement with *F*_ST_ values of 1.3% (Dadi et al., 2008) and 1.1% (Zerabruk et al., 2011) reported for Ethiopian indigenous populations in a study using microsatellite markers. However, it was lower than previously reported values for West African cattle breeds (6%; Ibeagha-Awemu and Erhardt, 2005), six African cattle breeds (4%; Gautier et al., 2007), Ankole cattle (4.6%; Kugonza et al., 2011), and Burlina cattle (8.5%; Dalvit et al., 2008). The level of within-population genetic variation was higher than that reported in Ankole cattle populations (95.54%; Kugonza et al., 2011) and Indian zebu breeds (88.7%; Mukesh et al., 2004). Within-population inbreeding (*F*_IS_) value of -0.003 and total inbreeding (*F*_IT_) value of 0.006 determined in this study are higher than values reported for Ethiopian indigenous cattle breeds based on microsatellite analyses (Dadi et al., 2008). The absence of any significant inbreeding effects may be a reflection of the high gene flow between the populations, as supported by high *N*_m_ values, the large population from which the samples were drawn, and the fact that related individuals were purposely avoided.

Based on genetic distance and genetic differentiation estimates, the populations under investigation are very closely related, in agreement with previous microsatellite-based investigations (Dadi et al., 2008; Zerabruk et al., 2011). The values representing genetic variation between Ethiopian and Hanwoo populations obtained here were close to those obtained in a previous study comparing African and European cattle breeds (15.5%; Gautier et al., 2007) and the F_CT_ value obtained between *B. indicus* and *B. taurus* subspecies (0.19; McKay et al., 2008). Number of migrants per generation (*N*_m_) values indicates the relative strength of gene flow and genetic drift. Genetic differentiation will result in substantial differentiation where *N*_m_ < 1 but not where *N*_m_ > 1 (Slatkin, 1987). In this investigation, the estimated number of migrants (*N*_m_) was considerably higher than an earlier estimate for Ankole cattle populations (Kugonza et al., 2011) and that among Indian *B. indicus* breeds (Mukesh et al., 2004) and signifies considerable gene flow between populations, resulting in low measures of genetic differentiation and inbreeding.

#### Structure analysis and PCA

The likelihood values and variance of the bootstrap were plotted against *K* values to select the optimum *K* values to provide the most reliable results. We found that the variance of likelihood increased slightly from *K *= 2 to 5 and reached its peak at *K* = 6, while the probability of the LnP (D) dramatically decreased beyond at the assumed *K* = 6. At *K *= 4 and 5, breeds or populations sampled from highland agro-ecology (Ambo, Arsi, and Horro) were grouped together, while lowland breeds (Borana and Danakil) tended to cluster separately at *K* = 6 and showed some degree of admixture. We found that the allelic frequencies for two of the highland populations were very similar (0.77 ± 0.15 and 0.78 ± 0.15 for Arsi and Ambo, respectively). This was further confirmed by a low *F*_ST_ value for these populations. The clustering of Borana and Danakil separately from other Ethiopian cattle populations could be attributed to their unique genetic compositions, geographical isolation, and ecological differences. The detected signature of admixture of the two breeds and separation from the rest of the groups could be due to the fact that, ancestrally, the two breeds share input from long horn taurus and *B. indicus* populations. Similarly, Danakil (sanga) is an intermediate type, formed by hybridization of the indigenous humpless cattle with zebu (Rege, 1999). These findings are also in agreement with the hypothesis that livestock facing selection pressure from environmental conditions, such as drought, are expected to show higher genomic divergence across habitats, compared to a neutral genome background (Hanotte et al., 2010). The grouping of Horro (sanga × zebu) with Arsi and Ambo is likely to be due to a high level of admixture and similarity of production environments. Phylogenetic, principal component and STRUCTURE analyses clearly separated the Ethiopian cattle populations from the Hanwoo breed, which is in accordance with their separate geographical origins, domestication, and divergence long before domestication of *B. indicus* and *B. taurus* subspecies (Loftus et al., 1994, 1999; Bradley et al., 1996). Further, the high divergence of Hanwoo cattle from Ethiopian cattle is consistent with the hypothesis of local domestication in Asia (McKay et al., 2008) and recent reports of independent domestication in Africa (Hanotte et al., 2002). The lack of clear differentiation between Ethiopian cattle populations according to their conventional classification is in a good agreement with already established facts showing that following the introduction of the zebu breeds on coast and Horn of Africa, there has been extensive hybridization between the zebu and original African long horn taurine cattle (Epstein, 1971; Rege, 1999; Hanotte et al., 2002; Freeman et al., 2006). The present study was also in line with the separate clustering of *B. indicus* and *B. taurus* breeds from principal component, phylogenic, and STRUCTURE analysis (Lin et al., 2010).

In conclusion, a significant amount of genetic variation is retained within indigenous Ethiopian cattle populations. Genetic distance, phylogenetic tree, principal component, and population structure analyses clearly differentiated the cattle populations according to their historical origins and represented the genetic distinctiveness of Ethiopian cattle populations from the Hanwoo breed. The high within-population genetic diversity and the unique adaptation of the current populations to wider environmental factors (disease, heat stress, drought, and feed shortage), might be a consequence of the peculiar admixture between the different cattle breeds. Hitherto, these populations have represented a unique genetic resource and unexploited opportunity that warrants initiatives for their sustainable conservation and utilization. The clustering of populations by ecological distribution is an insight suggesting that further investigation of the association between genetic markers and geo-environmental parameters could better enable exploitation of valuable genetic material. Apparent partition of the populations (Hanwoo and Ethiopian) according to their historical origins, and corroboration of the established details of the genetic diversity and composition of Ethiopian cattle populations, suggest that analyses using 4235 SNP markers provided sufficient genetic information to properly assess the genetic structure.

## Conflict of Interest Statement

The authors declare that the research was conducted in the absence of any commercial or financial relationships that could be construed as a potential conflict of interest.

## References

[B1] Boichard D. Chung H. Dassonneville R. David X. Eggen A. Fritz S.(2012). Design of a bovine low-density SNP array optimized for imputation. *PLoS ONE* 7:e34130. 10.1371/journal.pone.0034130 PMC3314603 22470530

[B2] Bradley D. G. MacHugh D. E. Cunningham P. Loftus R. T. (1996). Mitochondria diversity and the origins of African and European cattle. *Proc. Natl. Acad. Sci. U.S.A.* 93 5131–5135 8643540 10.1073/pnas.93.10.5131 PMC39419

[B3] Carruthers C. R. Plante Y. Schmutz S. M. (2011). Comparison of Angus cattle populations using gene variants and microsatellites. *Can. J. Anim. Sci.* 91 81–85

[B4] Central Statistical Authority (CSA) (2008).*Ethiopian Statistical Abstract*. Addis Ababa: CSA.

[B5] Dadi H. Kim J. J. Yoon D. Kim K. S. (2012). Evaluation of single nucleotide polymorphisms (SNPs) genotyped by the Illumina Bovine SNP50K in cattle focusing on Hanwoo breed. *Asian-Aust. J. Anim. Sci.* 25 28–32 10.5713/ajas.2011.11232 PMC4092922 25049474

[B6] Dadi H. Tibbo M. Takahashi Y. Nomura K. Hanada H. Amano A. (2008). Microsatellite analysis reveals high genetic diversity but low genetic structure in Ethiopian indigenous cattle populations. *Anim. Genet.* 39 425–431 18565163 10.1111/j.1365-2052.2008.01748.x

[B7] Dadi H. Tibbo M. Takahashi Y. Nomura K. Hanada H. Amano T. (2009). Variation in mitochondrial DNA and maternal genetic ancestry of Ethiopian cattle populations. *Anim. Genet.* 40 556–559 19397526 10.1111/j.1365-2052.2009.01866.x

[B8] Dalvit C. De Marchi M. Dal Zotto R. Zanetti E. Meuwissen T. Cassandro M. (2008). Genetic characterization of the Burlina cattle breed using microsatellites markers. *J. Anim. Breed. Genet.* 125 137–144 18363979 10.1111/j.1439-0388.2007.00707.x

[B9] DNA Genotek Inc. (2012). *Performa-*gene•LIVESTOCK (PG-100). Available at: http://www.dnagenotek.com/US/products/PG100.html [accessed April 8, 2012]

[B10] Edea Z. Dadi H. Kim S. W. Dessie T. Kim K. S. (2012). Comparison of SNP variation and distribution in indigenous Ethiopian and Korean Cattle (Hanwoo) populations. *Genomics Inform.* 10 200–205 23166531 10.5808/GI.2012.10.3.200 PMC3492656

[B11] Epstein H. (1971). *The Origin of the Domestic Animals of Africa*. New York: African Publishing Corporation (APC)

[B12] Excoffier L. Laval G. Schneider S. (2005). Arlequin (version 3.0): an integrated software package for population genetics data analysis. *Evol. Bioinform. Online* 1 47–50 19325852 PMC2658868

[B13] Freeman A. R. Bradley D. G. Nagda S. Gibson J. P. Hanotte O. (2006). Combination of multiple microsatellite data sets to investigate genetic diversity and admixture of domestic cattle. *Anim. Genet.* 37 1–9 16441289 10.1111/j.1365-2052.2005.01363.x

[B14] Gautier M. Faraut T. Moazami-Goudarzi K. Navratil V. Foglio M. Grohs C. (2007). Genetic and haplotypic structure in 14 European and African cattle breeds. *Genetics* 177 1059–1070 17720924 10.1534/genetics.107.075804 PMC2034613

[B15] Golden Helix, Inc. (2012). *SNP & Variation Suite Manual*, Version 7.6.9. Available at:http://www.goldenhelix.com [accessed April 10, 2012]

[B16] Hanotte O. Bradley D. G. Ochieng J. W. Verjee Y. Hill E. W Rege J. E. O. (2002). African pastoralism: genetic imprints of origins and migrations. *Science* 296 336–339 11951043 10.1126/science.1069878

[B17] Hanotte O. Dessie T. Kemp S. (2010). Time to tap Africa’s livestock genomes. *Science* 328 1640–1641 20576875 10.1126/science.1186254

[B18] Ibeagha-Awemu E. M. Erhardt G. (2005). Genetic structure and differentiation of 12 African *Bos indicus* and *Bos taurus* cattle breeds, inferred from protein and microsatellite polymorphisms. *J. Anim. Breed. Genet.* 122 12–20 16130484 10.1111/j.1439-0388.2004.00478.x

[B19] Köhler-Rollefson I. Rathore H. S. Mathias E. (2009). Local breeds, livelihoods and livestock keepers’ rights in South Asia. *Trop. Anim. Health Prod.* 41 1061–1070 19031006 10.1007/s11250-008-9271-x

[B20] Kugonza D. R. Jianlin H. Nabasirye M. Mpairwe D. Kiwuwa G. H. Okeyo A. M.(2011). Genetic diversity and differentiation of Ankole cattle populations in Uganda inferred from microsatellite data. *Livest. Sci.* 135 140–147

[B21] Li M. H. Zerabruk M. Vangen O. Olsaker I. Kantanen J. (2007). Reduced genetic structure of north Ethiopian cattle revealed by Y-chromosome analysis. *Heredity* 98 214–221 17213865 10.1038/sj.hdy.6800931

[B22] Lin B. Z. Sasazaki S. Mannen H. (2010). Genetic diversity and structure in *Bos taurus* and *Bos indicus* populations analyzed by SNP markers. *Anim. Sci. J.* 81 281–289 20597883 10.1111/j.1740-0929.2010.00744.x

[B23] Liu K. Muse S. V. (2005). PowerMarker: integrated analysis environment for genetic marker data. *Bioinformatics* 21 2128–2129 15705655 10.1093/bioinformatics/bti282

[B24] Loftus R. T. Ertugrul O. Harba A. H. El-Barody M. A. MacHugh D. E. Park S. D.(1999). A microsatellite survey of cattle from a centre of origin: the Near East. *Mol. Ecol.* 8 2015–2022 10632853 10.1046/j.1365-294x.1999.00805.x

[B25] Loftus R. T. MacHugh D. E. Bradley D. G. Sharp P. M. Cunningham P. (1994). Evidence for two independent domestications of cattle. *Proc. Natl. Acad. Sci. U.S.A.* 91 2757–2761 8146187 10.1073/pnas.91.7.2757 PMC43449

[B26] McKay S. D. Schnabel R. D. Murdoch B. M. Matukumalli L. K. Aerts J. Coppieters W.(2008). An assessment of population structure in eight breeds of cattle using a whole genome SNP panel. *BMC Genetics* 9:37. 10.1186/1471-2156-9-37 PMC2408608 18492244

[B27] Ministry of Agriculture (MoA) (1998). *Agroecological Zones of Ethiopia*. Addis Ababa: Natural Resources Management and Regulatory Department, MoA

[B28] Mukesh M. Sodhi M. Bhatia S. Mishra B. P. (2004). Genetic diversity of Indian native cattle breeds as analysed with 20 microsatellite loci. *J. Anim. Breed. Genet.* 121 416–424

[B29] Nei M. (1987). *Molecular Evolutionary Genetics*. New York: Columbia University Press

[B30] Notter D. R. (1999). The importance of genetic diversity in livestock populations of the future. *J. Anim. Sci.* 80 1776–1785 10.2527/1999.77161x 10064028

[B31] Pariset L. Mariotti M. Nardone A. Soysal M. I. Ozkan E. Williams J. L.(2010). Relationships between Podolic cattle breeds assessed by single nucleotide polymorphisms (SNPs) genotyping. *J. Anim. Breed. Genet.* 127 481–488 21077972 10.1111/j.1439-0388.2010.00868.x

[B32] Payne W. J. A. Wilson T. R. (1999). *An Introduction to Animal Husbandry in the Tropics*. Oxford: Blackwell Science Ltd

[B33] Peakall R. Smouse P. E. (2006). GENALEX 6: genetic analysis in excel. Population genetic software for teaching and research. *Mol. Ecol. Notes* 6 288–295 10.1093/bioinformatics/bts460 PMC3463245 22820204

[B34] Pritchard J. K. Stephens M. Donnelly P. (2000). Inference of population structure using multilocus genotype data. *Genetics* 155 945–959 10835412 10.1093/genetics/155.2.945 PMC1461096

[B35] Rege J. E. O. (1999). The state of African cattle genetic resources I. Classification framework and identification of threatened and extinct breeds. *Anim. Genet. Resour. Informat.* 25 1–25

[B36] Rege J. E. O. Gibson J. P. (2003). Animal genetic resources and economic development: issues in relation to economic valuation. *Ecol. Econ.* 45 319–330

[B37] Reynolds J. Weir B. S. Cockerham C. C. (1983). Estimation of the coancestry coefficient: basis for a short-term genetic distance. *Genetics* 105 767–779 17246175 10.1093/genetics/105.3.767 PMC1202185

[B38] Slatkin M. (1987). Gene flow and the geographical structure of natural populations. *Science* 236 787–792 3576198 10.1126/science.3576198

[B39] Sneath P. H. A. Sokal R. R. (1973). *Numerical Taxonom*. San Francisco: W.H. Freedman and Company

[B40] Tamura K. Peterson N. Stecher G. Nei M. Kumar S. (2011). MEGA5. molecular evolutionary analysis using maximum likelihood, evolutionary distance, and maximum parsimony methods. *Mol. Biol. Evol.* 28 2731–2739 10.1093/molbev/msr121 PMC3203626 21546353

[B41] Vignal A. Milan D. Sancristobal M. Eggen A. (2002). A review on SNP and other types of molecular markers and their use in animal genetics. *Genet. Sel. Evol*. 34 275–305 12081799 10.1186/1297-9686-34-3-275 PMC2705447

[B42] Weir B. S. Cockerham C. C. (1984). Estimating F-statistics for the analysis of population structure. *Evolution* 38 1358–1370 10.1111/j.1558-5646.1984.tb05657.x 28563791

[B43] Zerabruk M. Li M. H. Kantanen J. Olsaker I. Ibeagha-Awemu E. M. G. Erhardt G.(2011). Genetic diversity and admixture of indigenous cattle from North Ethiopia: implications of historical introgressions in the gateway region to Africa. *Anim. Genet.* 43 257–266 22486496 10.1111/j.1365-2052.2011.02245.x

